# Methods of electroencephalographic signal analysis for detection of small hidden changes

**DOI:** 10.1186/1753-4631-1-9

**Published:** 2007-07-28

**Authors:** Hiie Hinrikus, Maie Bachmann, Jaan Kalda, Maksim Sakki, Jaanus Lass, Ruth Tomson

**Affiliations:** 1 Department of Biomedical Engineering, Techomedicum of the Tallinn University of Technology, Tallinn, Estonia; 2 Department of Mechanics, Institute of Cybernetics at the Tallinn University of Technology, Tallinn, Estonia

## Abstract

The aim of this study was to select and evaluate methods sensitive to reveal small hidden changes in the electroencephalographic (EEG) signal. Two original methods were considered.

Multifractal method of scaling analysis of the EEG signal based on the length distribution of low variability periods (LDLVP) was developed and adopted for EEG analysis. The LDLVP method provides a simple route to detecting the multifractal characteristics of a time-series and yields somewhat better temporal resolution than the traditional multifractal analysis.

The method of modulation with further integration of energy of the recorded signal was applied for EEG analysis. This method uses integration of differences in energy of the EEG segments with and without stressor.

Microwave exposure was used as an external stressor to cause hidden changes in the EEG. Both methods were evaluated on the same EEG database. Database consists of resting EEG recordings of 15 subjects without and with low-level microwave exposure (450 MHz modulated at 40 Hz, power density 0.16 mW/cm^2^). The significant differences between recordings with and without exposure were detected by the LDLVP method for 4 subjects (26.7%) and energy integration method for 2 subjects (13.3%).

The results show that small changes in time variability or energy of the EEG signals hidden in visual inspection can be detected by the LDLVP and integration of differences methods.

## 1. Background

Analysis of dynamics of the electroencephalographic (EEG) signal is complicated due to the irregular nature of the signal. It is difficult to detect small variations in the EEG signals on the background of their high natural variability.

Achievements in EEG analysis have made it possible to distinguish between the disturbed states of a brain due to a strong stressor. Various methods can be used to evaluate the depth of anaesthesia [1], to detect physiological disorders in brain in epilepsy [2-5], to distinguish among sleep stages [6,7], etc. In many cases, the analysis by non-linear methods has proved useful. For example, Lopes da Silva et al. propose that neuronal networks involved in epilepsy possess multistable dynamics, which can be characterised in phase-space with different attractors [4]. It has also been demonstrated that entropy measures and correlation dimensions are useful for anticipating seizures [5].

The effect caused by a nonspecific weak stressor, such as a low-level microwave radiation or mental task, on the EEG activity, is usually very small and linear statistical analysis is unable to provide a reliable and statistically significant distinction between the EEG signals with and without the stressor [8]. This is one of the reasons why the question of whether any feasible effect of a low-level radiation on brain's bioelectric activity exists is still open. The difficulties in interpretation of the experimental results cause doubt in these effects. Despite extensive research in this field during recent decades the reports of possible effects are often contradictory and the mechanisms behind the effects are still unclear.

Our previous attempts on detection of the effect of microwave radiation on human EEG showed that some traditional methods of EEG analysis such as weighted spectral power of the EEG frequency bands, bispectrum or fractal dimension, usually successfully applied, did not provide reliable distinction of small changes in the EEG caused by the microwave radiation [9,10].

The EEG analysis using non-linear methods can be more sensitive with respect to small changes in the signals. Indeed, bioelectric signals are generated by a simultaneous activity of multiple sources modulated by different physiological factors, which are intermittent by their nature. Therefore, EEG signal can also be expected to be non-Gaussian and intermittent. Such intermittency can severely lower the stationarity of the signal; various non-linear measures have been devised to cope with intermittency and non-stationarity in the best possible way.

A multifractal method for the EEG analysis – scaling analysis of length distribution of low variability periods (LDLVP) was applied in this study. The scaling of the LDLVP has proven a sensitive tool for the multifractal interpretation of heart rate variability [11]. The first attempt of adaptation of the LDLVP for EEG analysis has been promising [12]. The LDLVP analysis provides a simple route to detecting the multifractal characteristics of a time-series and yields somewhat better temporal resolution than the traditional multifractal analysis [13-15]. Thus, it can be expected that this method is sensitive with respect to small "hidden" changes in such a complicated physiological signal as EEG.

In the case of detection of weak signals hidden in noise the method of modulation of the signal with further integration of energy is expected to be fruitful. The method of modulation has been proven as a sensitive tool for detection of nonspecific signals (changes in energy) in radio engineering. In this study such approach of integration of differences in energy between the signal segments with and without expected change was applied for the EEG analysis.

The study is aimed on experimental evaluation of the selected LDLVP and integration of differences methods on the same EEG database recorded in conditions of the exposure to microwave radiation. The hypothesis is that exposure to microwave radiation causes changes in time variability and energy of the recorded EEG signals. The radiation is assumed to produce instantaneous effect on the brain bioelectrical activity and the recorded EEG signal.

Estimation of individual sensitivity of subjects to microwave exposure provides testing of ability of the methods to reveal small changes in EEG and adds useful knowledge about microwave effect.

## 2. Method

### Database

#### 2.1. Subjects

An experimental study was carried out on a group of volunteers. The group consisted of 15 young persons (aged 21–24): 8 male and 7 female. Their physical and mental condition (tiredness, sleepiness) before the experiment was evaluated by a questionnaire and a clinical interview. All the subjects selected were healthy, without any medical or psychiatric disorders. Persons who declared tiredness or sleepiness before the experiment were excluded. After the recordings, they described how they felt during the experiment. The subjects reported neither alertness nor any strain experienced during the recordings.

The experiments were conducted with the understanding and written consent of each subject. The study was conducted in accordance with the Declaration of Helsinki and formally approved by the local Medical Research Ethics Committee.

The measurements were performed in a dark laboratory, but no other special conditions were provided. The subjects lay in a relaxed position, with eyes closed and ears blocked during the experiments.

All the subjects were exposed and sham exposed. Only one experimental EEG recording was performed for a subject during a day. The measurements were double blinded. During each test session, the exposed and sham-exposed subjects were randomly assigned. The subjects were not informed of their exposure; however, they were aware of the possibility of being exposed.

##### Microwave Exposure

The modulated microwave radiation at the non-thermal level of field power density, identical to our previous studies [16,17], was used. Microwave exposure conditions were the same for all subjects.

The 450 MHz microwave radiation was generated by the Rhode & Swartz (Germany) signal generator model SML02. The RF signal was 100% pulse modulated by the modulator SML-B3 at 40 Hz frequency (duty cycle 50%). The generator signal was amplified by the Dage Corporation (USA) power amplifier model MSD-2597601. Located in the laboratory, the generator and amplifier were carefully shielded. The 1W microwave output power was guided by a coaxial lead to the 13 cm rod antenna NMT450 RA3206 by Allgon Mobile Communication AB, Sweden, located 10 cm from the subject's skin on the left side of head.

The Central Physical Laboratory of the Estonian Health Protection Inspection measured the spatial distribution of the microwave power density by the Fieldmeter C.A 43 Chauvin Arnoux (France) field strength meter. The calibration curves of the field power density dependence on the distance from the radiating antenna were obtained from these measurements taken in the actual conditions of the experiment. During the experiments, the stability of the microwave level was monitored by the IC Engineering (USA) Digi Field C field strength meter. Estimated from the measured calibration curves, the field power density at the skin was 0.16 mW/cm^2^.

##### Recording protocols and equipment

**Figure 1:**
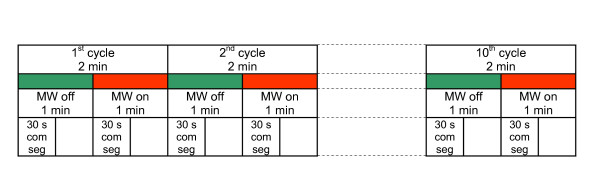
Time schedule of the recording protocol: 2 min cycles, 1 min reference and microwave half-periods, 30s comparison segments.

The study consisted of two experimental protocols, identical for all subjects. The first protocol is described below (Fig. 1).

First, the reference EEG was recorded over 60 s. Secondly, modulated at 40 Hz microwave radiation was applied. The duration of the exposure was also 60 s. Continuous EEG recordings were made during reference and microwave half-periods of the exposure cycle. The exposure cycle was repeated ten times. The recording protocol for one subject lasted for 20 min, during which the EEG was continuously recorded. Every odd minutes of the recordings (first half-periods of the cycles) were passive (the microwave exposure was switched off) and every even minutes of the recordings (second half-periods of the cycles) were active (the microwave exposure was switched on). During ten cycles of microwave exposure, the modulation frequency always remained at 40 Hz.

The second protocol for the sham-exposure included also the same steps, except that the microwave power was switched off. Odd and even minutes of the sham recordings were considered as reference and microwave half-periods of a cycle.

The Cadwell Easy II EEG measurement equipment was used for the EEG recordings. The EEG was recorded by means of 19 electrodes, placed on the subject's head according to the international 10–20-electrode position classification system, with Cz as reference. The EEG recordings were stored on a computer at a sampling frequency of 400 Hz. The 0.5 Hz high-pass and 70 Hz low-pass as well as 50 Hz notch hardware filters were used during recordings.

Pre-processing of the signal was performed in the LabVIEW programming and signal-processing environment. The EEG spectrum 0.5 – 48 Hz was selected for the further analysis by filtering. The modulation frequency 40 Hz was removed using a narrow-band filter. The signals bands of four basic EEG rhythm frequencies, theta (4 – 7 Hz), alpha (8 – 13 Hz), beta1 (15 – 20 Hz) and beta2 (22 – 38 Hz), were extracted from the total EEG signal by filtering. The elliptic bandstop filters with 50 dB attenuation in the stop-band were used.

An experienced neurologist examined the recorded EEG signals by visual inspection. Filtering while performing pre-processing of the signals reduced movement electromyographic artifacts but not cut off. The recordings with electrode artefacts were removed, and for these subjects the whole recording was repeated on another day.

The results of the preceding validation of the set-up on passive phantom confirmed the absence of modulation components, caused by parasitic interference between EEG and radio frequency equipment.

#### 2.2. Method: analysis of the EEG based on the LDLVP method

Initially, all the EEG recordings were divided into two sub-signals. The recordings performed with the first and second recording protocol were divided as follows:

• the first sub-signal contained all 1 min periods without microwave exposure (all the odd minutes from the initial EEG recording),

• the second sub-signal contained all minutes with microwave exposure (all even minutes of the initial EEG recording).

The recordings performed with the sham recording protocol were divided similarly: the first sham sub-signal contained all the odd minutes and the second sham sub-signal contained all the even minutes of the initial recording.

The scaling analysis utilizing LDLVP method was applied for two sub-signals.

**Figure 2:**
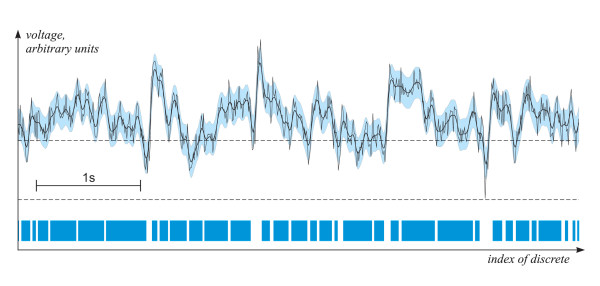
Scheme of the LDLVP method: thin line – recorded EEG signal (amplitude in arbitrary units); bold line – local average in time window *T*; blue zone – threshold value of the local variability *δ*_0_; line below – continuous intervals of the low-variability periods.

The LDLVP analysis consists of several steps (Fig. 2).

Firstly, we define the local average (bold line in Fig. 2) of the signal (thin line in Fig. 2) in time-window *T*

V(t)¯=1n∑r=1nV(r),
 MathType@MTEF@5@5@+=feaafiart1ev1aaatCvAUfKttLearuWrP9MDH5MBPbIqV92AaeXatLxBI9gBaebbnrfifHhDYfgasaacH8akY=wiFfYdH8Gipec8Eeeu0xXdbba9frFj0=OqFfea0dXdd9vqai=hGuQ8kuc9pgc9s8qqaq=dirpe0xb9q8qiLsFr0=vr0=vr0dc8meaabaqaciaacaGaaeqabaqabeGadaaakeaadaqdaaqaaiabdAfawjabcIcaOiabdsha0jabcMcaPaaacqGH9aqpdaWcaaqaaiabigdaXaqaaiabd6gaUbaadaaeWbqaaiabdAfawjabcIcaOiabdkhaYjabcMcaPaWcbaGaemOCaiNaeyypa0JaeGymaedabaGaemOBa4ganiabggHiLdGccqGGSaalaaa@40C8@

where *V*(r) is the amplitude of the recorded signal in a sample *r *and *n *is the number of samples in a time-window *T*. The time-window width *T *is a free (adjustable) parameter. The choice of the time-window is critical and guided by the following considerations. The lower limit should not be smaller than the dominant time-scale of high-frequency variations. The higher limit should not be too large, because *T *plays the role of the lower cut-off scale of LDLVP and the scaling range of LDLVP becomes too narrow. In order to achieve the widest possible scaling range, we opted for the smallest sensible value *T *= 60 ms. The number of samples in time-window *n *= 24.

Secondly, we define the local variability as the deviation of the current value of the signal from the local average.

δV(t)=V(t)−1n∑r=1nV(r).
 MathType@MTEF@5@5@+=feaafiart1ev1aaatCvAUfKttLearuWrP9MDH5MBPbIqV92AaeXatLxBI9gBaebbnrfifHhDYfgasaacH8akY=wiFfYdH8Gipec8Eeeu0xXdbba9frFj0=OqFfea0dXdd9vqai=hGuQ8kuc9pgc9s8qqaq=dirpe0xb9q8qiLsFr0=vr0=vr0dc8meaabaqaciaacaGaaeqabaqabeGadaaakeaaiiGacqWF0oazcqWGwbGvcqGGOaakcqWG0baDcqGGPaqkcqGH9aqpcqWGwbGvcqGGOaakcqWG0baDcqGGPaqkcqGHsisldaWcaaqaaiabigdaXaqaaiabd6gaUbaadaaeWbqaaiabdAfawjabcIcaOiabdkhaYjabcMcaPaWcbaGaemOCaiNaeyypa0JaeGymaedabaGaemOBa4ganiabggHiLdGccqGGUaGlaaa@47AC@

The threshold value of the local variability *δ*_0 _is determined (blue zone in Fig. 2).

Thirdly, the low-variability periods (intervals of the lower separate line in Fig. 2) are defined as continuous intervals with

*δV *(*t*) <*δ*_0_.

Finally, the number of low-variability periods *N *exceeding the length *T*_0 _is plotted versus the length *T*_0_.

The character of this length-distribution depends qualitatively on the threshold parameter *δ*_0_: if *δ*_0 _is very small, all the low-variability periods are very short; if *δ*_0 _is very large, there is a single low-variability period occupying the whole recording. For intermediate values of *δ*_0_, the non-trivial scale-invariant distribution law is observed [18,19]. In this study, the value of *δ*_0 _was adjusted for each recording individually, reaching a minimal value that for both sub-signals the length of the longest low-variability period was at least 3750 ms.

The hypothesis of this work assumes that microwave exposure causes change in the EEG variability. Due to higher variability, there are fewer long low-variability periods and vice versa. Therefore, it is expected that microwave exposure lowers or raises the curve at the right-hand part of the graph (i.e. at large values of *T*_0_). According to this presumption, the weighted area

SW=∑N=1128ln⁡(Nmax⁡(N−1,1/4))ln⁡(T0)N1/2
 MathType@MTEF@5@5@+=feaafiart1ev1aaatCvAUfKttLearuWrP9MDH5MBPbIqV92AaeXatLxBI9gBaebbnrfifHhDYfgasaacH8akY=wiFfYdH8Gipec8Eeeu0xXdbba9frFj0=OqFfea0dXdd9vqai=hGuQ8kuc9pgc9s8qqaq=dirpe0xb9q8qiLsFr0=vr0=vr0dc8meaabaqaciaacaGaaeqabaqabeGadaaakeaacqWGtbWudaWgaaWcbaGaem4vaCfabeaakiabg2da9maaqahabaGagiiBaWMaeiOBa42aaeWaaeaadaWcaaqaaiabd6eaobqaaiGbc2gaTjabcggaHjabcIha4jabcIcaOiabd6eaojabgkHiTiabigdaXiabcYcaSiabigdaXiabc+caViabisda0iabcMcaPaaaaiaawIcacaGLPaaaaSqaaiabd6eaojabg2da9iabigdaXaqaaiabigdaXiabikdaYiabiIda4aqdcqGHris5aOGagiiBaWMaeiOBa4MaeiikaGIaemivaq1aaSbaaSqaaiabicdaWaqabaGccqGGPaqkcqWGobGtdaahaaWcbeqaaiabigdaXiabc+caViabikdaYaaaaaa@5586@

under the curve of the function *T*_0_*= T*_0_*(N) *was selected as the multifractal quantitative measure.

In the denominator of this formula, *N*-1 is substituted by max (*N*-1,1/4) because of a simple reason: to take into account the longest low-variability period (with *N *= 1) without divergence of the expression. The weighting factor *N*^1/2 ^was introduced to enhance the stationarity of the measure. Namely, the least stationary part of the *T*_0_*(N)*-curve is the region *N *≈ 1, because the relative statistical uncertainty of *N *at a given *T*_0 _is inversely proportional to the square root of the number of underlying data points *N*^-1/2^. The overall variance is minimised when each term of the sum has a weight equal to the reciprocal of its uncertainty.

#### 2.3. Method: Analysis of the EEG based on the integration of differences method

The EEG analysis was performed separately for each EEG rhythms frequency bands: theta (4 – 7 Hz), alpha (8 – 13 Hz), beta1 (15 – 20 Hz) and beta2 (22 – 38 Hz).

The method of integration of differences consisted of several steps.

Firstly, the average energy of the signal inside a selected comparison segment in a time-window *T *was calculated as

si=1n∑r=1n[V(r)]2
 MathType@MTEF@5@5@+=feaafiart1ev1aaatCvAUfKttLearuWrP9MDH5MBPbIqV92AaeXatLxBI9gBaebbnrfifHhDYfgasaacH8akY=wiFfYdH8Gipec8Eeeu0xXdbba9frFj0=OqFfea0dXdd9vqai=hGuQ8kuc9pgc9s8qqaq=dirpe0xb9q8qiLsFr0=vr0=vr0dc8meaabaqaciaacaGaaeqabaqabeGadaaakeaacqWGZbWCdaWgaaWcbaGaemyAaKgabeaakiabg2da9maalaaabaGaeGymaedabaGaemOBa4gaamaaqahabaWaamWaaeaacqWGwbGvdaqadaqaaiabdkhaYbGaayjkaiaawMcaaaGaay5waiaaw2faaaWcbaGaemOCaiNaeyypa0JaeGymaedabaGaemOBa4ganiabggHiLdGcdaahaaWcbeqaaiabikdaYaaaaaa@4167@

where *V*(*r*) is the amplitude of the recorded signal in a sample *r *and *n *is the number of samples in a time-window *T*.

The locations of the comparison segments in the exposure cycle and time-window width *T *are the free (adjustable) parameters.

The location of the comparison segments in active and passive parts of the exposure cycle should provide their maximal difference. The selection depends on physiological parameters of brain: reaction time to the exposure and adaptation time constant. The choice of the locations was done based on our previous experimental data for segments located in the beginning, middle and the end of the active and passive parts of the exposure cycle. Finally, the comparison segments were placed in the beginning of the active as well as passive parts of the exposure cycle.

The time-window width *T *must be large enough to minimize influence of the natural EEG fluctuations. The value of *T *should not be too large and not exceed the brain adaptation time constant. In this case the selected value of *T *was 30 s. During 30 seconds number of samples *n *= 12000.

Finally, the first 30 s intervals of 60 s recording half-cycles with and without exposure are selected as the signal segments for comparison.

Secondly, relative differences of the average energies for segments with and without stimulation were calculated for every cycle *f*:

Sf=(s2f−s1fs1f)×100%,
 MathType@MTEF@5@5@+=feaafiart1ev1aaatCvAUfKttLearuWrP9MDH5MBPbIqV92AaeXatLxBI9gBaebbnrfifHhDYfgasaacH8akY=wiFfYdH8Gipec8Eeeu0xXdbba9frFj0=OqFfea0dXdd9vqai=hGuQ8kuc9pgc9s8qqaq=dirpe0xb9q8qiLsFr0=vr0=vr0dc8meaabaqaciaacaGaaeqabaqabeGadaaakeaacqWGtbWudaWgaaWcbaGaemOzaygabeaakiabg2da9maabmaabaWaaSaaaeaacqWGZbWCdaWgaaWcbaGaeGOmaiJaemOzaygabeaakiabgkHiTiabdohaZnaaBaaaleaacqaIXaqmcqWGMbGzaeqaaaGcbaGaem4Cam3aaSbaaSqaaiabigdaXiabdAgaMbqabaaaaaGccaGLOaGaayzkaaGaey41aqRaeGymaeJaeGimaaJaeGimaaJaeiyjauIaeiilaWcaaa@4547@

where *s*_1f _and s_2f _are the average energies in a comparison segment without and with microwave respectively. Integration of the differences over ten cycles of exposure for a subject *m *was applied and characteristic parameter *S*_*m *_was calculated

Sm=110∑f=110s2f−s1fs1f.
 MathType@MTEF@5@5@+=feaafiart1ev1aaatCvAUfKttLearuWrP9MDH5MBPbIqV92AaeXatLxBI9gBaebbnrfifHhDYfgasaacH8akY=wiFfYdH8Gipec8Eeeu0xXdbba9frFj0=OqFfea0dXdd9vqai=hGuQ8kuc9pgc9s8qqaq=dirpe0xb9q8qiLsFr0=vr0=vr0dc8meaabaqaciaacaGaaeqabaqabeGadaaakeaacqWGtbWudaWgaaWcbaGaemyBa0gabeaakiabg2da9maalaaabaGaeGymaedabaGaeGymaeJaeGimaadaamaaqahabaWaaSaaaeaacqWGZbWCdaWgaaWcbaGaeGOmaiJaemOzaygabeaakiabgkHiTiabdohaZnaaBaaaleaacqaIXaqmcqWGMbGzaeqaaaGcbaGaem4Cam3aaSbaaSqaaiabigdaXiabdAgaMbqabaaaaaqaaiabdAgaMjabg2da9iabigdaXaqaaiabigdaXiabicdaWaqdcqGHris5aOGaeiOla4caaa@4859@

For sham recordings the same parameters were calculated for comparison segments inside even and odd minutes of the recordings.

The relative difference in the EEG energy between the recording segments with and without exposure was selected as a measure to detect effects for further statistical analysis.

#### 2.4. Statistical analysis

For sham recordings, signal segments with and without exposure are completely equivalent. The mathematical expectation of the difference in their energies is zero, ⟨*s*_*s*1 _- *s*_*s*2_⟩ = 0. Next, an estimate of the variance could be obtained as the mean of squared differences for sham recordings:

σ2=115∑m=115(SSm)2
 MathType@MTEF@5@5@+=feaafiart1ev1aaatCvAUfKttLearuWrP9MDH5MBPbIqV92AaeXatLxBI9gBaebbnrfifHhDYfgasaacH8akY=wiFfYdH8Gipec8Eeeu0xXdbba9frFj0=OqFfea0dXdd9vqai=hGuQ8kuc9pgc9s8qqaq=dirpe0xb9q8qiLsFr0=vr0=vr0dc8meaabaqaciaacaGaaeqabaqabeGadaaakeaaiiGacqWFdpWCdaahaaWcbeqaaiabikdaYaaakiabg2da9maalaaabaGaeGymaedabaGaeGymaeJaeGynaudaamaaqahabaWaaeWaaeaacqWGtbWudaWgaaWcbaGaem4uamLaemyBa0gabeaaaOGaayjkaiaawMcaamaaCaaaleqabaGaeGOmaidaaaqaaiabd2gaTjabg2da9iabigdaXaqaaiabigdaXiabiwda1aqdcqGHris5aaaa@41A4@

According to the "zero hypothesis", the EEG recordings of subjects under microwave exposure cannot be distinguished from sham signals. Thus, the "zero hypothesis" implies that ⟨*s*_1 _- *s*_2_⟩ = 0 and ⟨(*s*_1 _- *s*_2_)^2^⟩ = ⟨(*s*_*s*1 _- *s*_*s*2_)^2^⟩. Consequently, if the zero hypothesis is true, the quantity *x *= (*S*_*m*_)^2 ^*σ*^-2 ^is an *f*-distributed random quantity, the cumulative distribution of which is routinely designated as *F*_1,15_*(x)*; the indices 1 and 15 stand for the numbers of the degrees of freedom.

Accordingly, the ratio of the computed power difference to the standard deviation of the differences can be used as a quantitative measure, showing how well the zero hypotheses is satisfied; respective *p*-values are obtained by means of the cumulative *f*-distribution:

*p*_*m *_= *F*_1,15 _⌊(*S*_*m*_)^2 ^σ^-2^⌋

The same technique has been applied to the multifractal quantitative measure (derived from LDLVP), resulting in another series of *p*-values for microwave exposed and sham recordings.

For post hoc analysis the modified Bonferroni correction was applied according to which the smallest p-value is to be multiplied by the number of data points 15, the second smallest is to be multiplied by 15/2 = 7.5 etc.

## 3. Results

**Figure 3:**
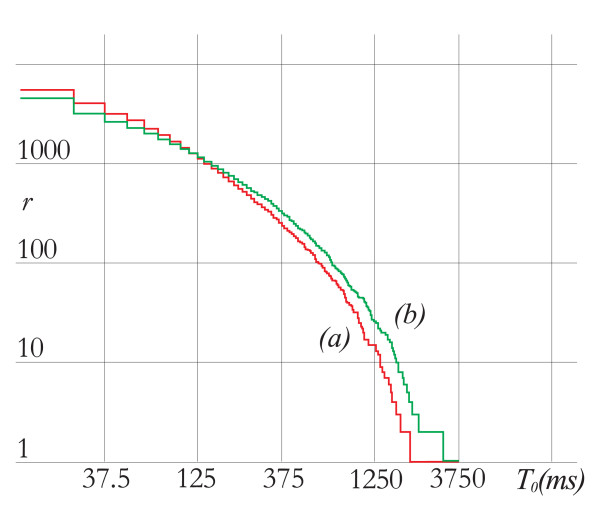
The number of low-variability periods *N *exceeding the length *T*_0 _for a significant subject: red line (a) – EEG signal with microwave (second sub-signal); green line (b) – EEG signal without microwave (first sub-signal).

The results of LDLVP analysis for a subject are presented in Figure 3. The number of low-variability periods *N *exceeding the length *T*_0 _is plotted versus the length *T*_0 _for the first and second sub-signal for exposed recording. As can be seen, microwave exposure lowers the curve at the right-hand part of the graph (large values of *T*_0_). Such a change in curve indicates that microwave exposure increases variability of the EEG signal: owing to higher variability there are fewer long low variability periods.

The results of statistical analysis of the LDLVP quantitative measures for sham and microwave-exposed recordings, calculated for each subject, are presented in Table 1. For sham recordings there were no significant results. The ratio of the computed power difference to the standard deviation of differences x
 MathType@MTEF@5@5@+=feaafiart1ev1aaatCvAUfKttLearuWrP9MDH5MBPbIqV92AaeXatLxBI9gBaebbnrfifHhDYfgasaacH8akY=wiFfYdH8Gipec8Eeeu0xXdbba9frFj0=OqFfea0dXdd9vqai=hGuQ8kuc9pgc9s8qqaq=dirpe0xb9q8qiLsFr0=vr0=vr0dc8meaabaqaciaacaGaaeqabaqabeGadaaakeaadaGcaaqaaiabdIha4bWcbeaaaaa@2E40@ of more than three were considered as significant deviations from the zero hypothesis and are marked in bold. After Bonferroni correction *p*-values not larger than 0.05 were considered as significant deviations from the zero hypothesis and are marked in bold. As can be seen, the analysis resulted in *p*-values lower than 0.05 for 4 cases in the case of microwave exposure. Results of analysis for the whole group were not statistically significant (*p *> 0.5).

**Table 1:** Analysis using the LDLVP method: calculated x
 MathType@MTEF@5@5@+=feaafiart1ev1aaatCvAUfKttLearuWrP9MDH5MBPbIqV92AaeXatLxBI9gBaebbnrfifHhDYfgasaacH8akY=wiFfYdH8Gipec8Eeeu0xXdbba9frFj0=OqFfea0dXdd9vqai=hGuQ8kuc9pgc9s8qqaq=dirpe0xb9q8qiLsFr0=vr0=vr0dc8meaabaqaciaacaGaaeqabaqabeGadaaakeaadaGcaaqaaiabdIha4bWcbeaaaaa@2E40@ and *p*-values as a result of Bonferroni correction for sham and microwave exposed (MW) conditions in P-channels (significant marked bold).

	x MathType@MTEF@5@5@+=feaafiart1ev1aaatCvAUfKttLearuWrP9MDH5MBPbIqV92AaeXatLxBI9gBaebbnrfifHhDYfgasaacH8akY=wiFfYdH8Gipec8Eeeu0xXdbba9frFj0=OqFfea0dXdd9vqai=hGuQ8kuc9pgc9s8qqaq=dirpe0xb9q8qiLsFr0=vr0=vr0dc8meaabaqaciaacaGaaeqabaqabeGadaaakeaadaGcaaqaaiabdIha4bWcbeaaaaa@2E40@		*p *– value	
Subject	sham	MW	sham	MW

1	0.07	0.95	1.000	0.591
2	-0.11	**-3.02**	0.582	**0.025**
3	1.02	1.30	1.000	0.392
4	0.81	-0.21	0.876	0.835
5	0.67	-0.39	0.963	0.812
6	0.81	-0.28	0.844	0.838
7	0.98	-0.63	0.837	0.802
8	-1.23	-1.51	0.804	0.315
9	1.86	**3.69**	0.715	**0.022**
10	-0.28	2.49	0.769	0.065
11	0.88	0.46	1.000	0.816
12	0.00	**3.09**	1.000	**0.029**
13	-0.04	1.93	1.000	0.170
14	-1.30	**-3.69**	1.000	**0.011**
15	1.93	0.49	1.000	0.857

**Figure 4:**
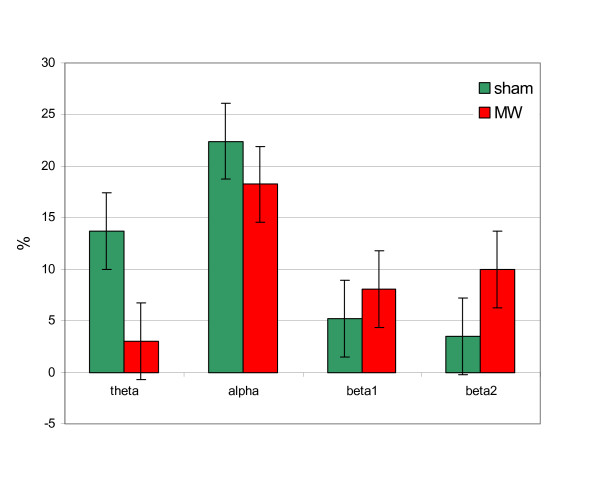
The relative average changes of the EEG rhythms energy of the segments with and without microwave exposure in P – channels for the whole group for microwave exposed (MW) and sham recordings.

The results of the *S *– parameter analysis for the whole group are presented in Fig. 4. The graph illustrates the effect of microwave exposure – differences between exposed and not exposed segments of the recordings for different EEG rhythms. As can be seen, microwave exposure causes increase in energy of the EEG beta1 and beta2 rhythms.

The results of statistical evaluation of the *S *– parameter for different subjects are presented in Table 2. For sham recordings there were no significant results. Microwave exposed recordings at modulation frequency 40 Hz have 2 significant values in beta2 region. Analysis for the whole group didn't reveal statistical significance.

**Table 2:** Analysis using the method of integration of differences: calculated *p*-values for different EEG rhythms in P-channels as a result of Bonferroni correction for sham and microwave exposed (MW) conditions (significant marked bold).

Frequency band	*p*-value							
	
	Theta		Alpha		Beta1		Beta2	
Subject	sham	MW	sham	MW	sham	MW	sham	MW

1	1.000	1.000	1.000	1.000	1.000	0.433	0.935	1.000
2	1.000	1.000	0.612	1.000	0.902	0.664	0.780	**0.000**
3	0.342	1.000	1.000	1.000	0.876	1.000	0.903	0.880
4	1.000	1.000	1.000	1.000	0.983	1.000	0.875	1.000
5	1.000	1.000	1.000	1.000	0.819	1.000	0.780	1.000
6	1.000	1.000	1.000	1.000	0.859	1.000	0.905	1.000
7	1.000	1.000	1.000	1.000	0.780	1.000	0.781	1.000
8	1.000	1.000	1.000	1.000	0.767	1.000	1.000	1.000
9	1.000	1.000	1.000	1.000	0.680	1.000	0.836	0.988
10	1.000	1.000	1.000	1.000	0.837	0.609	0.800	0.595
11	0.151	1.000	1.000	1.000	1.000	1.000	1.762	1.000
12	1.000	1.000	1.000	1.000	0.705	1.000	0.837	1.000
13	0.548	1.000	0.077	1.000	0.794	1.000	0.757	1.000
14	1.000	1.000	1.000	1.000	1.000	0.383	0.935	**0.003**
15	1.000	1.000	1.000	1.000	1.000	1.000	1.000	1.000

**Figure 5:**
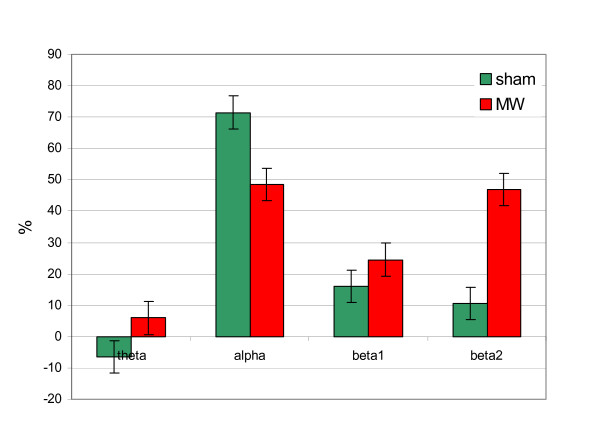
The relative changes of the EEG rhythms energy of the segments with and without microwave exposure in P – channels for a significant subject for microwave exposed (MW) and sham recordings.

The graphs of changes of the EEG rhythms energy for a significant subject are presented in Fig. 5. In this case increase in the EEG beta2 rhythm energy is more clearly indicated than in Fig. 4 for average changes in the whole group.

## 4. Discussion

The results show that small changes in the EEG signals hidden in visual inspections can be detected by the LDLVP and integration of differences methods.

As Table 1 illustrates, the LDLVP analysis presents the outcome, resulting in significant results for 4 subjects. Accordingly, significant effect of exposure to the EEG signal was detected for 26.7 % of subjects.

Considering the direction of influence at modulation frequency 40 Hz, for two subjects under the exposure the computed LDLVP weighted area increased, and for two it decreased. For all these subjects, the departure from the sham behavior was statistically reliable. Our previous study at modulation frequency 217 Hz resulted with the outcome, in which for half of the subjects the computed LDLVP weighted area decreased and for another half it decreased [12]. These observations give us a hint that the effect of the microwave stimulation on EEG time variability is different for different subjects.

The *S*-parameter measures exceeded the limit of significant deviation from zero hypothesis in beta2 frequency band (Table 2), providing 2 significant cases out of 15, that is 13.3 %.

Analysis with the integration of differences method revealed increase of the EEG energy in beta rhythm caused by microwave exposure. Increased beta absolute power was also observed in alcohol-dependent subjects [20]. The increased beta power in the resting EEG may be an electrophysiological index of the imbalance in the excitation-inhibition homeostasis in the cortex [20].

Statistical analysis didn't reveal significant effect of microwave for the whole group. The reason is very high variability among individual EEG signals as well as different individual sensitivity to microwave. Differences between the microwave stimulation and sham were statistically insignificant for the whole group also in our previous study at 7 Hz modulation frequency [17]. However, there were significant differences in some channels within individual subjects.

From Tables 1 and 2, it can be seen that the subjects having significant results overlap. On the other hand, with the LDLVP method, there are 4 significant results compared to 2 with the *S*-parameter method. This indicates that microwave stimulation causes different effects for different subjects and there is an obvious need for various methods to detect those effects.

From the results presented it is difficult to conclude which measure, LDLVP or *S*-parameter, is more effective and whether the effect appear rather in intensity (*S*-parameter) or time variability (LDLVP) of the EEG signals.

The analysis by the LDLVP and the integration of differences methods detected the effect of exposure at modulation frequency 40 Hz for 26.7 and 13,3 % of subjects. This value is even higher than the rate of multiple chemical sensitivity (MCS) occurrences that is estimated to be between 2 and 10 % in the general population [21]. MCS is characterized by recurrent symptoms involving multiple organ systems and occurring in response to demonstrable exposures to multiple chemically unrelated compounds at doses far below those established to cause harmful effects. Taking this into consideration, LDLVP and *S*-parameter methods demonstrated good sensitivity detecting the effects of microwave exposure.

## Authors' contributions

HH carried out design and coordination of the study, developed the integration of differences method for the EEG analysis. MB participated in the design of the study, carried out analysis of the EEG based on the integration of differences method, participated in statistical analysis and EEG recordings. JK developed the LDLVP method, participated in the EEG analysis based on the LDLVP method. MS carried out analysis of the EEG based on the LDLVP method and statistical analysis. JL participated in the design of the study and analysis of the EEG based on the integration of differences method. RT performed statistical analysis and participated in the EEG recordings. All authors read and approved the final manuscript.
